# Mesoscopic and macroscopic investigation of a dolomitic marble subjected to thermal damage

**DOI:** 10.1038/s41598-022-19655-x

**Published:** 2022-09-12

**Authors:** Jian-bin Liu, Zhong-jian Zhang, Anthony Kwan Leung

**Affiliations:** 1 Department of Civil Engineering, School of Engineering and Technology, China University of Geosciences (Beijing), No. 29 Xueyuan Road, Haidian District, Beijing, 100083 China; 2 Department of Civil and Environmental Engineering, School of Engineering, The Hong Kong University of Science and Technology, Clear Water Bay, Hong Kong SAR China

**Keywords:** Solid Earth sciences, Engineering, Materials science, Physics

## Abstract

Thermal loading is an important factor that could lead to the weakening and deterioration of rock materials. Understanding the thermal properties of rocks and their evolution under different high temperatures is important in the post-fire-hazard evaluation and cultural heritage conservation. Yet it is challenging to understand the evolution of thermally-induced changes in rock properties and to quantitatively study degrees of thermal damage when samples are limited. This study investigates the effects of high temperatures (i.e., 200 °C, 400 °C, 600 °C, 800 °C, and 1000 °C) on a dolomitic marble using combined mesoscopic and macroscopic testing techniques. The test results show that increasing marble temperature led to a deterioration of physical properties (i.e., increasing open porosity and weight loss; but decreasing P-wave velocity) and mechanical properties (i.e., increasing axial strain corresponding with the peak stress; but decreasing uniaxial compressive strength, Young’s modulus, and brittleness). There existed a threshold temperature of 600 °C, which marks different thermal damage mechanisms. Below the threshold, the rock deterioration was mainly caused by physical changes such as crack propagation and grain breakage, which can be characterized by mesoscopic parameters (i.e., linear crack density and mineral grain size distribution). On the contrary, when the temperature was higher than the threshold, the deterioration was caused by chemical changes, including mineral decomposition and re-crystallization, which was indicated by the changes in mineral compositions and relative atomic mass calculation. Based on the experimental results (e.g., mineralogical and physico-mechanical changes) and obtained relationships between the parameters in mesoscale and macroscale, a novel scheme for thermal damage evaluation is proposed to estimate thermally-induced changes in macroscopic parameters (e.g., Young’s modulus) based on the corresponding mesoscopic parameters (e.g., particle size distribution and linear crack density).

## Introduction

Thermal loading is an important factor that could lead to the weakening and deterioration (i.e., known as thermal damage) of rocks used as construction materials. For example, fires can lead to severe damage to masonry structures such as historical buildings and cultural heritage sites^1^. A previous study has pointed out that, on average, one historical structure was ruined per day by fire in European Union^2^. In China, the most important heritage site, the Forbidden city (classified as a UNESCO World Heritage site in 1987) has gone through several severe fires in history^3^. Since high temperature could lead to irreversible damage to these structures, studying the effects of thermal loading on rock materials and developing relevant methods to evaluate the thermal damage plays an important role in proper post-fire-hazard evaluation, restoration technique development, and conservation of masonry structures in cultural heritage sites^4^.

Thermal loading on rock materials is a process that influences the rock pore properties at the mesoscopic level and then the mechanical engineering behaviour at the macroscopic level^5–8^. Studying the evolution of mesoscopic properties of rocks such as thermally-induced changes in the types and amounts of rock-forming minerals, grain sizes of minerals and crack initiation, generation and propagation are vital to understand and interpret any deterioration of macroscopic mechanical behavior of rocks^9–12^. On a mesoscopic scale, there are three factors that are known to account for the thermal deterioration of rocks: anisotropic thermal expansion ratios of rock-forming minerals^13,14^, mineral decomposition and recrystallization (or phase change)^15^, and thermal gradient inside rock samples (i.e. thermal shock) during heating and cooling processes^16,17^. These deterioration processes due to thermal loading provide an important and scientific basis to study the thermal effects on the changes in physical and mechanical properties of rocks.

In recent years, a vast volume of research has been conducted to study the influences of high temperature on the properties of various rock types. These studies revealed that the high-temperature treatments have significant effects on rock behaviour at both macroscopic and mesoscopic levels. After exposure to temperatures higher than a critical point (e.g., 200 °C), some macroscopic physical properties (e.g., bulk density, porosity, hardness, ultrasonic velocities)^2,9,18–20^ and mechanical properties (e.g., uniaxial compressive strength and Young’s modulus)^4,10,21,22^ of rocks generally reduced. The initiation and propagation of intergranular and intragranular micro-cracks had also been identified^7,23,24^. In fact, the mesoscopic and macroscopic properties are intrinsically related. For example, the initiation and propagation of micro-cracks lengthened the transmission paths of P-wave and decreased P-wave velocity^9^; the development of larger amounts of micro-cracks can lead to a stronger non-linearity of the stress–strain curve in crack closure stage during compression^25^, hence decrease the peak strength and elastic modulus by facilitating crack growth and crack coalesce in later compression stages^26^.

In most of these studies, mainly macroscopic properties such as strength and the corresponding strain were investigated. Mesoscopic properties of rocks (e.g., crack density and particle size of mineral grains) are often only used to provide a qualitative explanation for the observed changes in the macroscopic properties^17,27,28^. The evolution of mesoscopic properties due to the changes in temperature is not clear, and the corresponding quantitative analyses to explain the macroscopic behaviour of rocks under different temperatures are limited. Any trans-scale (i.e., mesoscopic-macroscopic) relationship in different high temperatures is yet to be studied. Methods to classify the degree of influence of high temperatures on engineering applications are very limited.

The main objectives of this paper are to investigate the thermal effects on the mesoscopic properties and their evolution under different temperatures and to seek for the correlations with the macroscopic properties. In this laboratory study, dolomitic marble samples were heated to different temperatures of a wide range (i.e., up to 1000 °C) to simulate thermal damage since the thermal-related problems such as fire hazard effects may involve high temperatures up to 900 °C–1000 °C^29,30^. Any thermally-induced changes in the mesoscopic properties (i.e., size distributions of micro-pores, particle size distribution of minerals, number of micro-cracks, micro-crack density) and macroscopic properties (i.e., weight loss, open porosity, P-wave velocity, complete uniaxial stress–strain relationships) were systematically measured. The mesoscopic and macroscopic properties were parameterized. Attempts were made to explore the temperature dependence of the physical and mechanical parameters from mesoscopic perspectives and then to seek any correlations between the macroscopic and mesoscopic parameters. Based on the test results, a novel scheme of thermal damage evaluation of rock is proposed.

## Results

### Mineral compositions

The mineral compositions of the tested marble and their variation with the increase in maximum temperatures are presented in Table 1. Before being subjected to thermal treatments, the marble, which is regarded as a single-phase rock in this study, is mostly composed of dolomite mineral (98.6%) with small amounts of quartz (0.4%) and clay minerals (1.0%).

**Table 1:** Mineralogical compositions of marble samples exposed to different temperatures.

Samples	Mineralogical compositions (unit, wt%)					
	Quartz	Calcite	Dolomite	Lime	Periclase	Clay minerals
M25	0.4	–	98.6	–	–	1.0
M200	0.7	–	98.8	–	–	0.5
M400	0.6	–	98.4	–	–	1.0
M600	2.0	1.4	95.1	–	–	1.5
M800	2.1	65.1	–	–	32.8	–
M1000	–	–	–	85.0	15.0	–

Normally decimal part of XRD data only has qualitative meaning, decimal part in this table is self-generated by software.

As shown in Table 1, there is no significant mineralogical phase change before heating up to 400 °C. There was limited decomposition of dolomite mineral at 600 °C, and a small quantity of calcite was found. When subjected to higher temperatures, however, significant phase change occurred; the dolomite mineral has been completely decomposed and partially recrystallized. The minerals of marble exposed to 800 °C transformed into calcite (65.1%) and periclase (32.8%), while the samples exposed to 1000 °C transformed into lime (85.0%) and periclase (15.0%). The clay minerals in these marble samples all disappeared after exposing to 800 °C and 1000 °C.

### Mercury intrusion porosimetry

**Figure 1:**
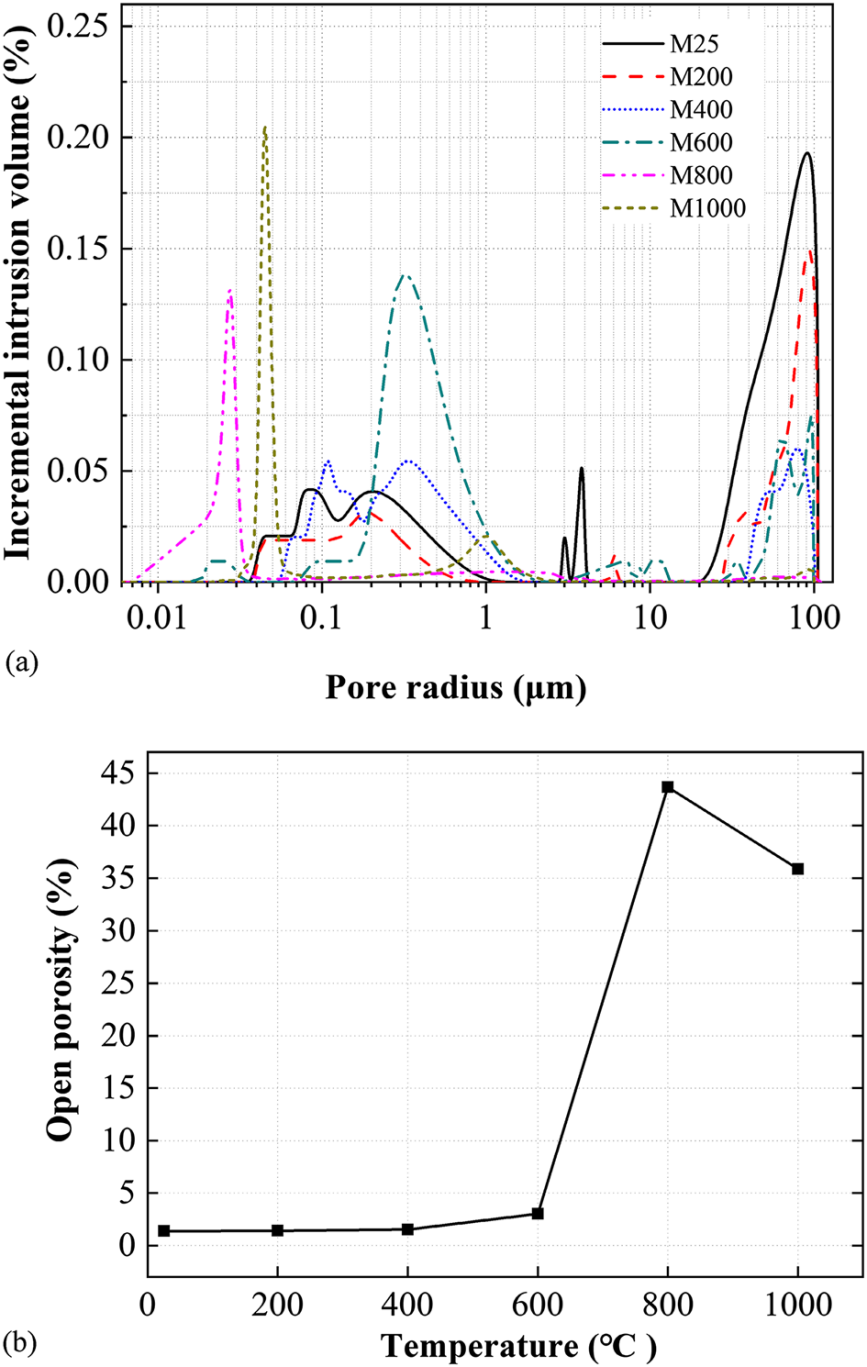
(**a**) Pore size distribution and (**b**) open porosity of marble samples exposed to different temperatures.

The results of the mercury intrusion porosimetry (MIP) tests are presented in Fig. 1. In general, the pore size distribution peaks of the rock samples shifted from bigger to smaller radii (i.e., from bigger micro-cracks to smaller micro-cracks) as the temperature increased. Interpreting the pore size distribution results with the porosity data (also obtained from the MIP tests), the shift was mainly attributed to the increase in smaller micro-cracks caused by the thermally-induced crack initiation and propagation, and the increase in smaller micro-cracks is faster than the increase in bigger micro-cracks. For instance, the volume of mercury intruded into the pores with radii greater than 10 μm for M25 and M1000 was 0.0023 cc/g and 0.0041 cc/g, respectively. It is clear that although the percentage of cumulative volume intruded in the pores of M1000 (1.72%) was lower than that of the M25 (42.95%), the absolute volume of pores in the M1000 sample was higher than that in M25.

It is also evident in Fig. 1a that the evolution of micro pore size distribution under different temperatures can be classified into three stages: (a) stage one: initial stage (i.e., M25 to M400); (b) stage two: transitional stage (i.e., M600); and (c) stage three: saltational stage (i.e., M800 and M1000). In stage one, the pore size distribution concentrated at the two ends of the spectrum. The percentage of bigger pores (> 10 μm) decreased, while the other end (0.1–1 μm) showed a trend of shifting towards smaller pore radii. Stage two is defined as the transition stage; this is, the bigger pore size shrank to the range of 40–100 μm, while the percentage of smaller pores increased significantly and concentrated at approximately 0.4 μm, indicating a significant development of micro-pores after exposure to 600 °C. In stage three, the number of micro-cracks rapidly increased due to the mineral decomposition and gas emission (Table 1) and entrapped gas expansion. The micro-pores smaller than 0.06 μm dominated the total pores. After exposing to 800 °C and 1000 °C, the pores centred within the range of 0.02–0.03 μm and 0.04–0.05 μm, respectively. Figure 1b shows the open porosity of the tested MIP samples. There was almost no change of porosity when the temperature treatment was less than 400 °C. Beyond this threshold, the porosity started increasing significantly by 12 times when exposed to 800 °C. Interestingly, the porosity of M1000 showed a drop of 9% compared with that of M800. This is related to the merging of grain boundaries that shrank the relatively bigger pores and wider cracks, supported by the grain image analysis later.

### Optical microscopy

**Figure 2:**
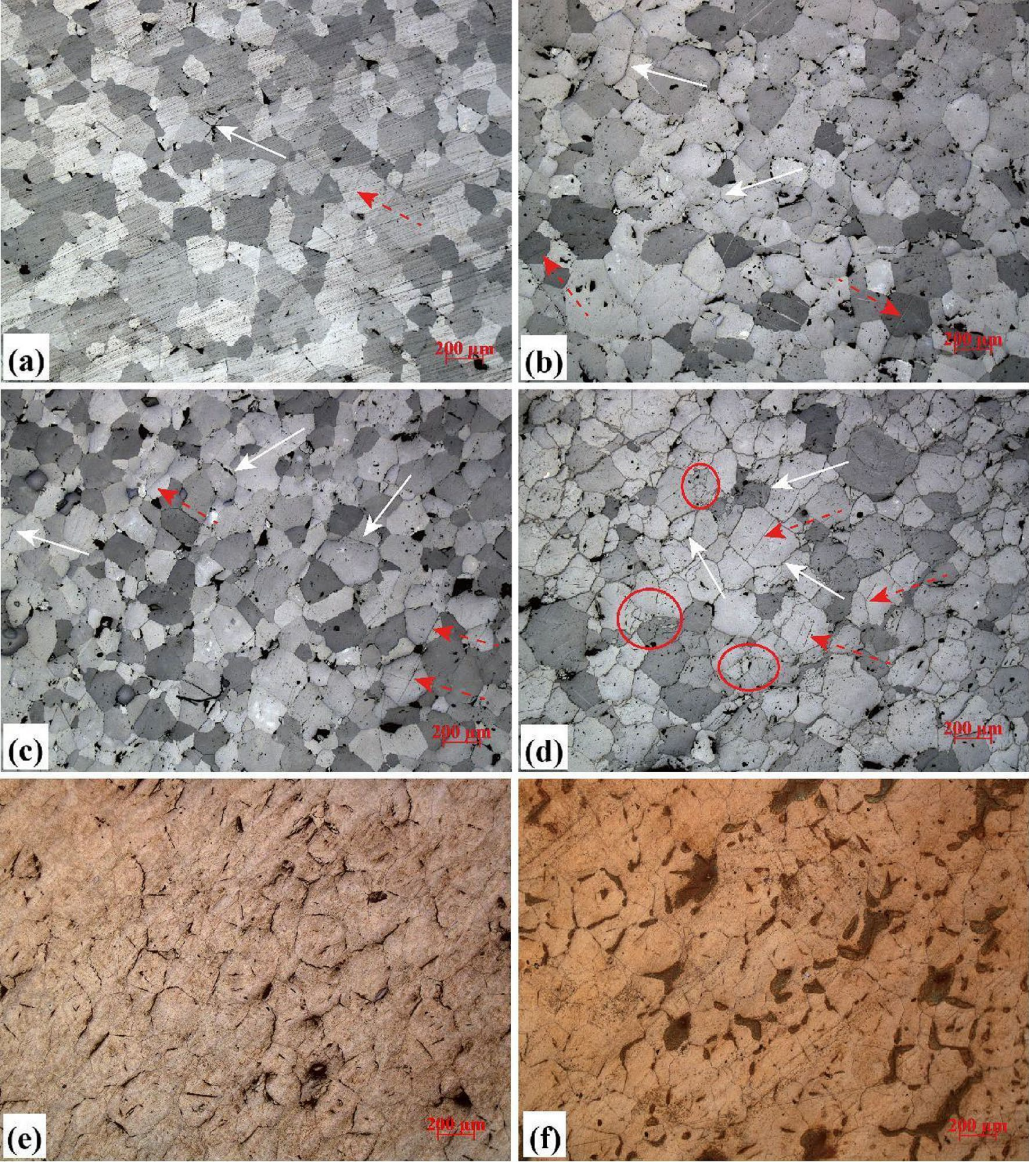
Photomicrographs of marble samples exposed to different temperatures in plane-polarized light: (**a**) M25; (**b**) M200; (**c**) M400; (**d**) M600; (**e**) M800; and (**f**) M1000. From photo (**a**–**d**), the inter-granular micro-cracks (indicated by white solid arrows) and intra-granular micro-cracks (indicated by red dotted arrows) are developed more extensively as the increase of treatment temperature. Crush zones (indicated by red circles) are observed in photo (**d**).

The results of optical microscopic observation tests shown in Fig. 2 reveal that marble samples subjected to higher treating temperature developed more micro-cracks, smaller grain sizes, and more complex mesoscopic properties. Before thermal treatments, a few micro-pores (point-like black areas) and very few micro-cracks (wire-like black areas) were observed (Fig. 2a). After heated to 200 °C, the marble showed an increase in both inter-granular micro-cracks and intra-granular micro-cracks (Fig. 2b). When heated to 400 °C, there was prominent and extensive propagation of inter-granular cracks and the initiation of intra-granular micro-cracks became widespread (Fig. 2c). After being heated to 600 °C, both of the inter-granular and intra-granular micro-cracks were well developed (Fig. 2d). The cementation among individual grains was almost destroyed, while the cracks appear to be widened compared with the crack size found in the samples subjected to lower temperature. Intra-granular micro-cracks were so well-developed that even multiple micro-cracks could be found in a single mineral grain. In some areas where the local thermal stress exceeds the strength of mineral grains adjacent to each other, crush zones were formed (indicated by circles in the figure). For M600, almost all of the grain boundaries were detached. Intra-granular cracks were well-developed, but no sign of color or texture change was observed (Fig. 2d). It is noteworthy that the development of micro-cracks below 600 °C was mainly caused by the inconsistent thermal expansion and contraction of the compositional minerals, since the temperatures applied were lower than the decomposition temperature of the major rock-forming mineral (i.e., dolomite; above 700 °C)^31^. In this stage, although the micro-structures of the samples changed (e.g., development of inter-granular and intra-granular micro-cracks, grain crush, etc.) with the magnitude of temperature exposed, the mineral composition remained practically the same.

A different phenomenon occurred when the temperature applied exceeded the decomposition temperature of the dolomite mineral. Significant crack development, color change and texture change of mineral grains were observed in M800 and M1000 samples (Fig. 2e,f). After heated to 800 °C, the decomposition process had directly led to the development of micro-cracks. The connectivity of the micro-crack network appears to be enhanced. Interestingly, after heated to 1000 °C, the edge of grain boundaries became less obvious, and some micro-cracks seemed to be healed compared with the case of M800.

In general, the optical microscopy results are consistent with the MIP results shown in Fig. 1. The more significant development of thermally-induced micro-cracks (Fig. 2) led to an increase in open porosity (Fig. 1b) when the treatment temperature was higher. Also, the increase in smaller cracks or pores shown in Fig. 1a is the result of micro-crack initiation and propagation, as shown in Fig. 2. The micro-crack development was limited when the marbles were heated to lower than 400 °C (Fig. 2a–c); by contrast, the inter-granular and intra-granular micro-cracks were well-developed in M600. The extensive micro-cracks from decomposition and phase change features of M800 and M1000 were likely responsible for the porosity increase depicted in Fig. 1. As presented in Fig. 2e,f, the trace of melting, merging and re-crystallization of minerals in M1000 may be responsible for the drop of open porosity in comparison with M800 (refer to Fig. 1). The two independent test results in Figs. 1 and 2 suggest that the existence of melting and merging of grain boundaries in 1000 °C treated sample may reduce the width (and probably number) of wider micro-cracks (e.g., the “shift” of bigger micro-cracks in Fig. 1a), and thus make the material less porous compared with the samples treated in 800 °C (e.g., the 9% drop of the open porosity shown in Fig. 1b).

### Weight loss

**Figure 3:**
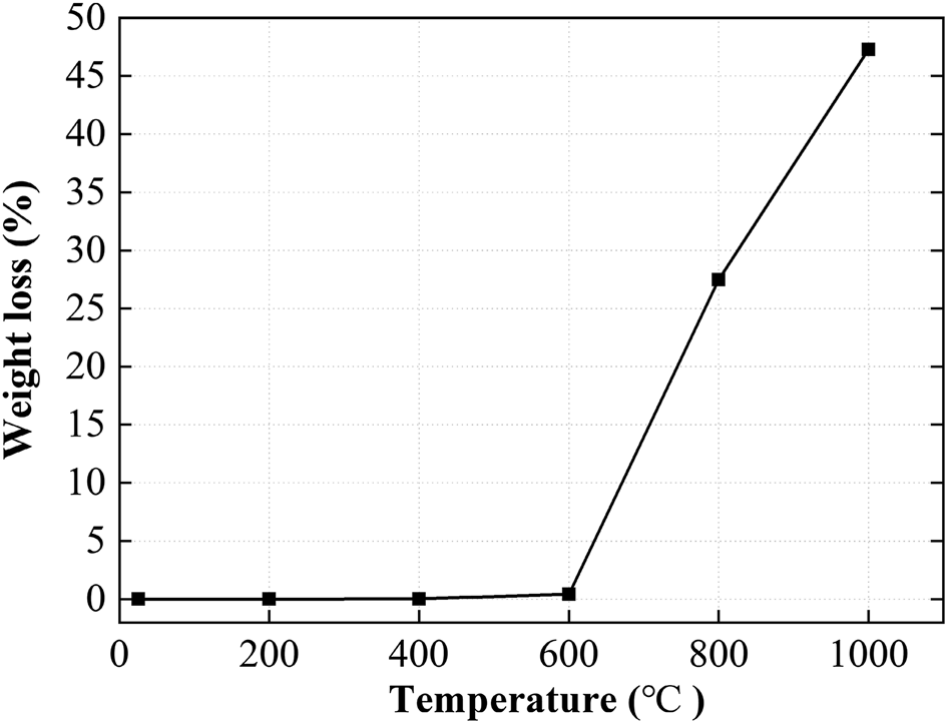
Measured weight loss of the marble samples exposed to different treating temperatures.

Figure 3 shows the effects of temperature treatments on the weight loss of the marble samples. It is evident that the marble started to lose its weight only until the temperature treatment reached beyond 400 °C. While there was very limited weight lost (i.e., 0.43%) from M400 to M600, a further increase in temperature treatment to 1000 °C resulted in almost 50% of weight loss (with respect to the case of M25). The weight loss was attributed to the mineral decomposition at different treating temperatures and may be explained by the following physiochemical processes. Previous studies have shown that dolomite would decompose into carbonates of magnesium (MgCO_3_) and calcium (CaCO_3_) when the temperature exceeds 700 °C^31^. While the MgCO_3_ would decompose into MgO when the temperature exceeds 500 °C, the calcite mineral would decompose into lime (CaO) when the temperature ranges between 850 and 925 °C^32^. Hence, the reaction processes under 800 °C and 1000 °C may be described by the following chemical equations:

When subjected to 800 °C,

1 $${\text{MgCa}}\left( {{\text{CO}}_{{3}} } \right)_{{{2}({\text{s}})}} = {\text{ CaCO}}_{{{3}({\text{s}})}} + {\text{ MgO}}_{{({\text{s}})}} + {\text{CO}}_{{{2}({\text{g}})}}$$

When subjected to 1000 °C,

2 $${\text{MgCa}}\left( {{\text{CO}}_{{3}} } \right)_{{{2}({\text{s}})}} = {\text{ CaO}}_{{({\text{s}})}} + {\text{ MgO}}_{{({\text{s}})}} + {\text{ 2CO}}_{{{2}({\text{g}})}}$$

The relative atomic mass of Ca, Mg, O, and C is 40, 24, 16, and 12, respectively. Based on these masses, the percentage of gas evaporated in Eqs. (1) and (2) after reaction can be calculated as 23.9% and 47.8%, respectively. These results are consistent with the weight loss of sample treated at 800 °C (weight loss = 27.5%) and 1000 °C (weight loss = 47.3%). The calculated results are also consistent with the XRD results presented in Table 1, which shows that M800 consisted of 65% calcite (CaCO_3_) and 32% periclase (MgO) and that M1000 consisted of 85% lime (CaO) and 15% periclase. The formation and escaping process of carbon dioxide (CO_2_) is one of the major reasons causing the observed micro-crack developing in Fig. 2.

### P-wave velocity

**Figure 4:**
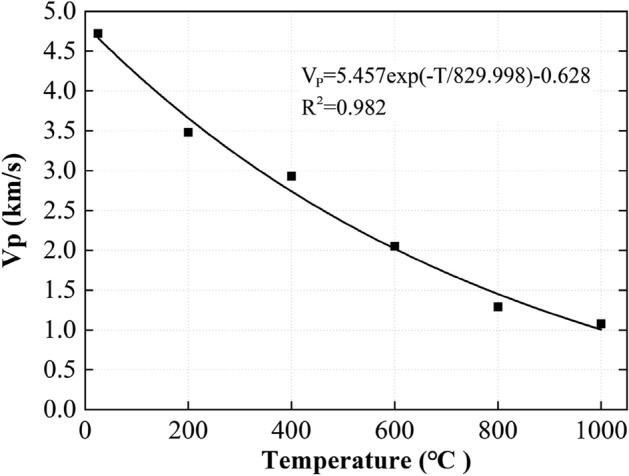
Relationships between P-wave velocity, V_p_, and maximum treating temperature, T.

The P-wave velocity, V_p_, is a parameter that could indicate thermal damage of rocks^33,34^. Figure 4 depicts the relationship of V_p_ and treating temperature. It is evident that V_p_ reduced exponentially with temperature, following almost a negative power law, which is consistent with previous research^10^. The change of V_p_ (Fig. 4) was negatively related to the change of open porosity (Fig. 1). As the temperature was higher, more micro-cracks and micro-pores were generated, and therefore the path length of the ultrasonic wave was also increased. The V_p_ dropped by 73.9% from the peak of 4.6 km/s when the treating temperature increased from 25 to 1000 °C. These observations reveal that V_p_ excited in the same frequency was sensitive to the micro-cracks and mineralogical phases of the samples. The relatively large drop of V_p_ between 25 and 200 °C was mainly caused by the initiation and propagation of micro-cracks. The further drop of V_p_ between 200 and 600 °C was comparatively less significant and were attributed to the propagation of micro-cracks (refer to Fig. 2b–d). Both the micro-crack development and the decomposition and re-crystallization of minerals were responsible for the further decrease of V_p_ between 600 and 1000 °C. Indeed, the open porosity of the sample treated at 800 °C was nine times the sample treated at 600 °C (Fig. 1b), yet the drop of V_p_ from 600 to 800 °C was similar to that from 400 to 600 °C. This implies that the influence of phase change of dolomite may have outweighed the effects of propagation of micro-cracks.

### Mechanical properties

**Figure 5:**
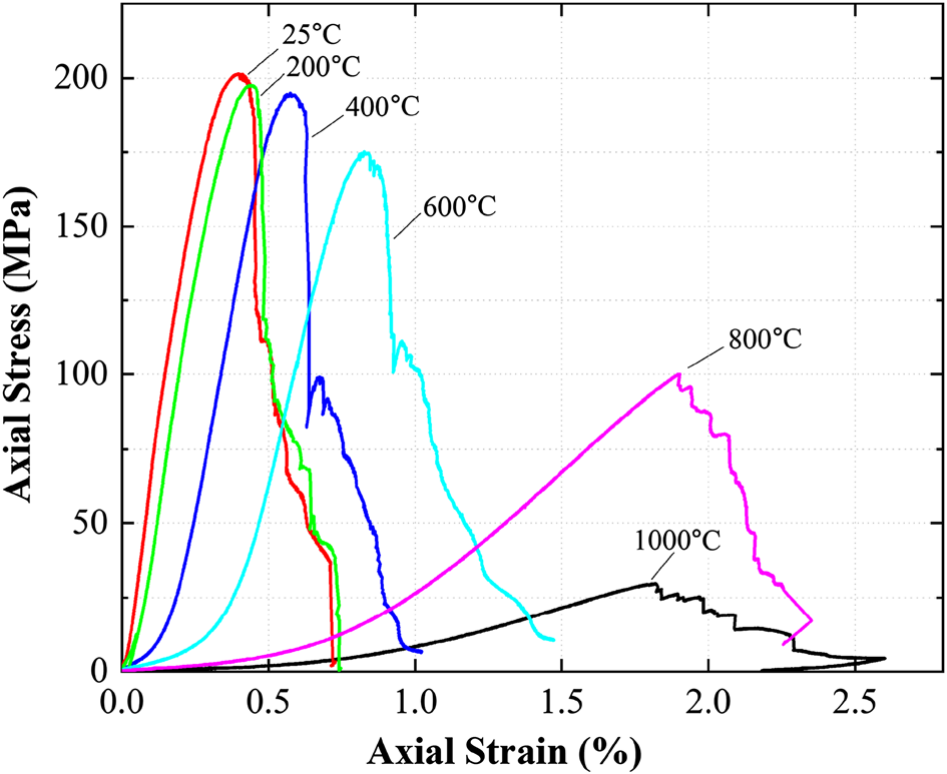
Stress–strain relationships of marble samples exposed to different treating temperatures.

Figure 5 shows the effects of treating temperature on the stress–strain curves obtained from the unconfined compression tests. Clearly, the treating temperature played a significant role in both the pre-peak and post-peak stages. When subjected to compression, the pores and any cracks in a rock would be gradually closing as the compressive force increases. This is represented by the initial curvy stage in all the stress–strain curves measured, typically known as the crack-closure stage. The threshold strain that the curve starts to become linear (i.e., the strain value obtained from extending the linear portion of the curve to the strain axis) is termed as crack-closure strain^25^. As the treating temperature increased, the crack-closure strain increased before the curve reached the linear stage (Fig. 5). The increase in the treating temperature also caused an evident reduction of the peak stress, while the strain corresponding to this peak stress increased. The test results also depict that the samples treated after 400 °C and 600 °C showed abrupt drops of stress in the post-peak stage^35^. Post peak, the mechanical behaviour of the marble may be primarily controlled by the slippage of the main crack surfaces of samples. The test results indicate that there may exist a threshold temperature between 600 and 800 °C, below which brittle failure mode prevailed. On the contrary, when the treating temperature was above the threshold, the marble became more ductile, and the abrupt post-peak drop of strength was less prominent.

**Figure 6:**
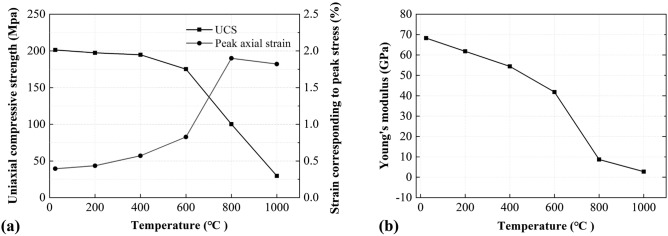
Effects of treating temperature on the mechanical properties of the marble samples: (**a**) uniaxial compressive strength and strain corresponding to peak stress, (**b**) Young’s modulus.

To further investigate the effects of treating temperature on the mechanical behaviour, the peak strength, Young’s modulus (i.e., the gradient of the linear portion of the curve, pre-peak) and the strain corresponding to the peak strength are correlated with the treating temperature in Fig. 6. In general, as the treating temperature increased, both the peak strength and Young’s modulus reduced while the strain corresponding to the peak strength increased. The thermal dependency of these three parameters may be classified into three stages: from 25 to 400 °C, from 400 to 600 °C, and from 600 to 1000 °C. The thermal effects were less significant in the first stage because of the limited development of micro-cracks. However, when reaching the second stage, the three parameters started to show some changes due to the micro-crack development. At the final stage, all parameters showed significant changes due to the decomposition and re-crystallization of the rock-forming mineral.

## Discussion

### Relationship between mesoscopic and macroscopic properties

The thermally-induced initiation and propagation of micro-cracks are irreversible and present a cumulative effect, as evidenced in Figs. 1 and 2. The magnitude of thermally-induced change in the mesoscopic properties of a rock governs its macroscopic properties^9,36,37^. Therefore, there is a need to study the trans-scale relationships between mesoscopic and macroscopic properties.

As discussed in the previous sections qualitatively, the thermally-induced mesoscopic changes were mainly associated with the initiation and propagation of micro-cracks, breakage of mineral grains, and decomposition and re-crystallization of minerals (i.e., phase change). Based on these understandings, the following method is used to evaluate the thermal damage quantitatively and to study any trans-scale relationships of the marble. The XRD data are used as the indicator of mineral phase change to determine the critical treating temperature. Before the critical temperature, the thermal damage was induced by grain breakages and micro-cracks. Thus, the grain size distribution and linear crack density are adopted as the quantitative mesoscopic indices to analyze the evolution of macroscopic mechanical properties of the rock after different temperatures treatments. The analyses only involved samples treated below the critical temperature (600 °C in the present study), because the types of rock-forming minerals were no longer the same after being treated above the critical temperature, as shown in Table 1 and Eqs. (1) and (2).

#### Grain size distributions-Rayleigh distribution

A grain size distribution of crystalline materials often follows lognormal or Rayleigh distribution^38^. Kumari et al.^39^ studied the crack distributions of granite using the Rayleigh distribution. Rong et al.^40^ directly recommended to use inter-granular and intra-granuar cracks as an index to study thermal effect on the performance of rock in a thermal field; these cracks affect the change of grain size distribution. In the present study, three photomicrographs are analyzed for the samples treated at each temperature. The grain sizes are extracted to evaluate their distribution characteristics, thermal dependence, and correlations with macroscopic properties. For each treating temperature, three images of the same size and amplification times were analyzed. A total of twelve images were used for statistical analysis.

**Figure 7:**
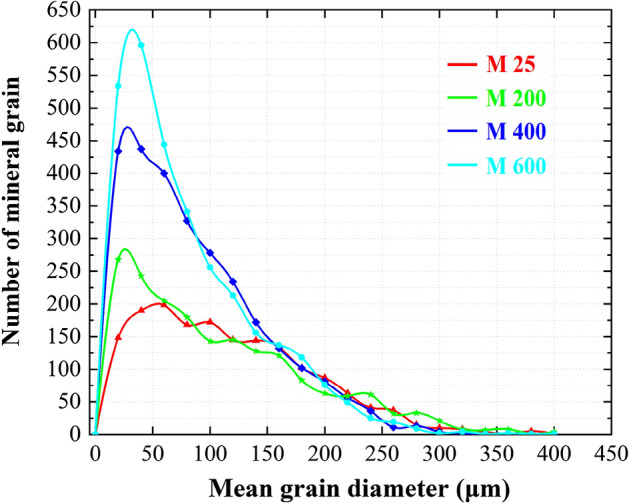
Evolution of grain size distributions of the marble samples exposed to different treating temperatures.

Figure 7 shows the grain size distributions of the marble samples treated at 25 °C, 200 °C, 400 °C and 600 °C. As the treating temperature increased, the number of smaller mineral grains increased significantly. The grain size distributions of the tested samples appear to follow the modified Rayleigh distribution (Supplementary figures):

3 $$f(x,\mu ) = a\frac{x}{{\mu^{2} }}e^{{ - \frac{{x^{2} }}{{2\mu^{2} }}}} ,(x \ge 0)$$

where a and μ denote the amplification parameter and the scale parameter, respectively.

**Figure 8:**
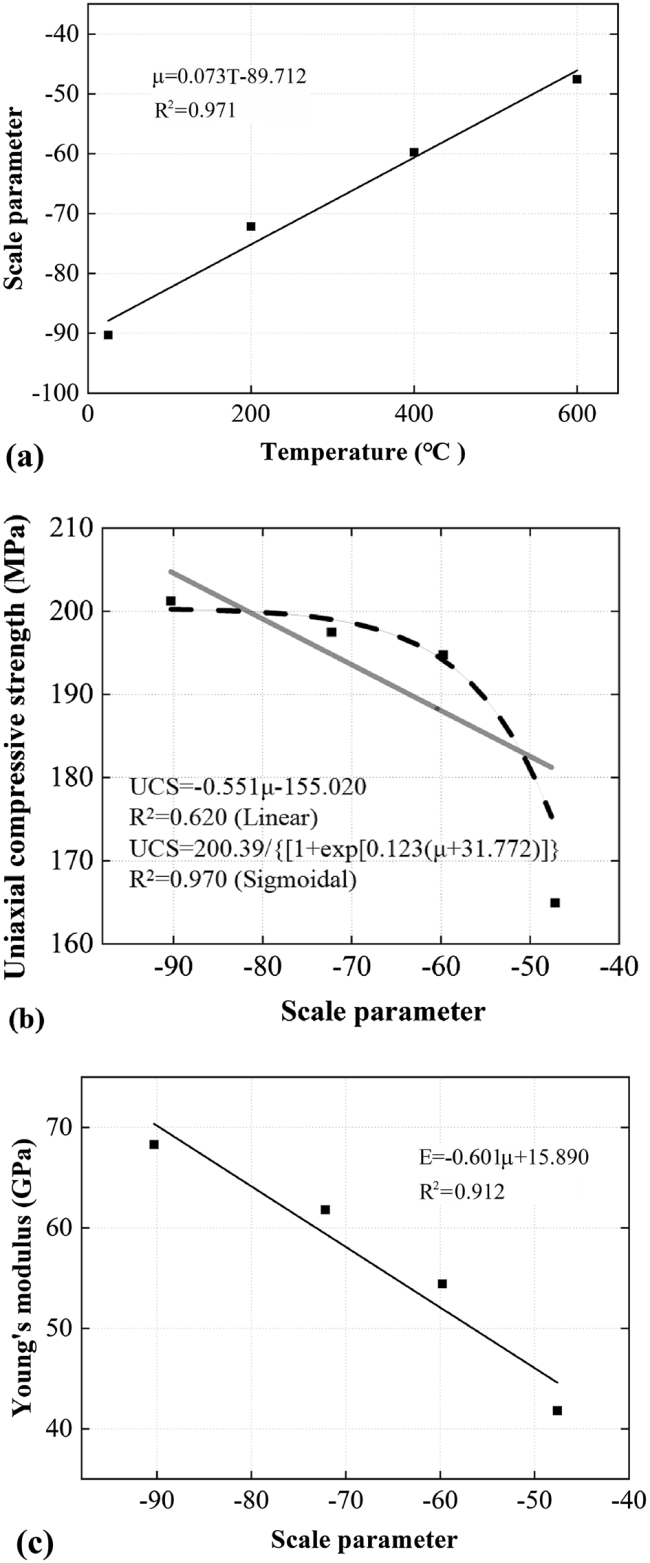
Correlations between (**a**) scale parameter and treating temperature, (**b**) UCS and the scale parameter and (**c**) E and the scale parameter.

The two fitting parameters of the modified Rayleigh distribution are summarized in Table 2 and related to the treating temperature in Fig. 8a. Evidently, the scale parameter has a strong and significant correlation with the treating temperature, apparently following a linear trend. The scale parameter is then correlated with the mechanical properties of the marble, including the UCS (Fig. 8a) and E (Fig. 8b). When samples were treated at higher temperatures, the thermally-induced crystal crushes were more intense. During this process, the breakage of bonding and the decrease of local load bearing capacity in the crush zone (marked in Fig. 2) led to the decrease in UCS and E; The increase in the number of smaller particles can reflect the thermally-induced increase in the number of local crush zones. The scale parameter, which reflects the mesoscopic behaviour of the marble, hence explained the thermally-induced changes in the macroscopic mechanical behaviour including UCS and E. Note that although better fitting can be achieved using negative exponential correlation in Fig. 8b and c and such relations have experimental evidence (e.g. the curves in Fig. 6a and b non-linearly bend down as treatment temperature linearly increase), as the pioneering investigation the present study use linear correlation to build relationships. When sufficient tests and data (at least dozens or hundreds of data) are available in the future, more accurate fitting correlations may be further built.

**Table 2:** Fitting parameters for the modified Rayleigh distribution and the corresponding goodness-of-fit at different treating temperatures.

Sample	Scale parameter (μ)	Amplification parameter (a)	R^2^
M25	− 90.299	30,308.588	0.854
M200	− 72.165	25,869.099	0.621
M400	− 59.755	45,585.798	0.837
M600	− 47.593	46,910.188	0.841

#### Linear crack density

Fresh crystalline rocks such as marble and granite are normally considered as “intact/compact” materials before thermal damage since there are limited pores and cracks^41^. The thermal damage of marble often displays in the form of micro-crack initiation and propagation. Linear crack density (ρ) is therefore used as a parameter to characterize the mesoscopic damage due to the thermal treatments^40^. This parameter is defined as:

4 $$\rho = \frac{L}{A}$$

where L and A denotes the total length of micro-cracks (as identified in an image) and the total area of the image, respectively.

**Figure 9:**
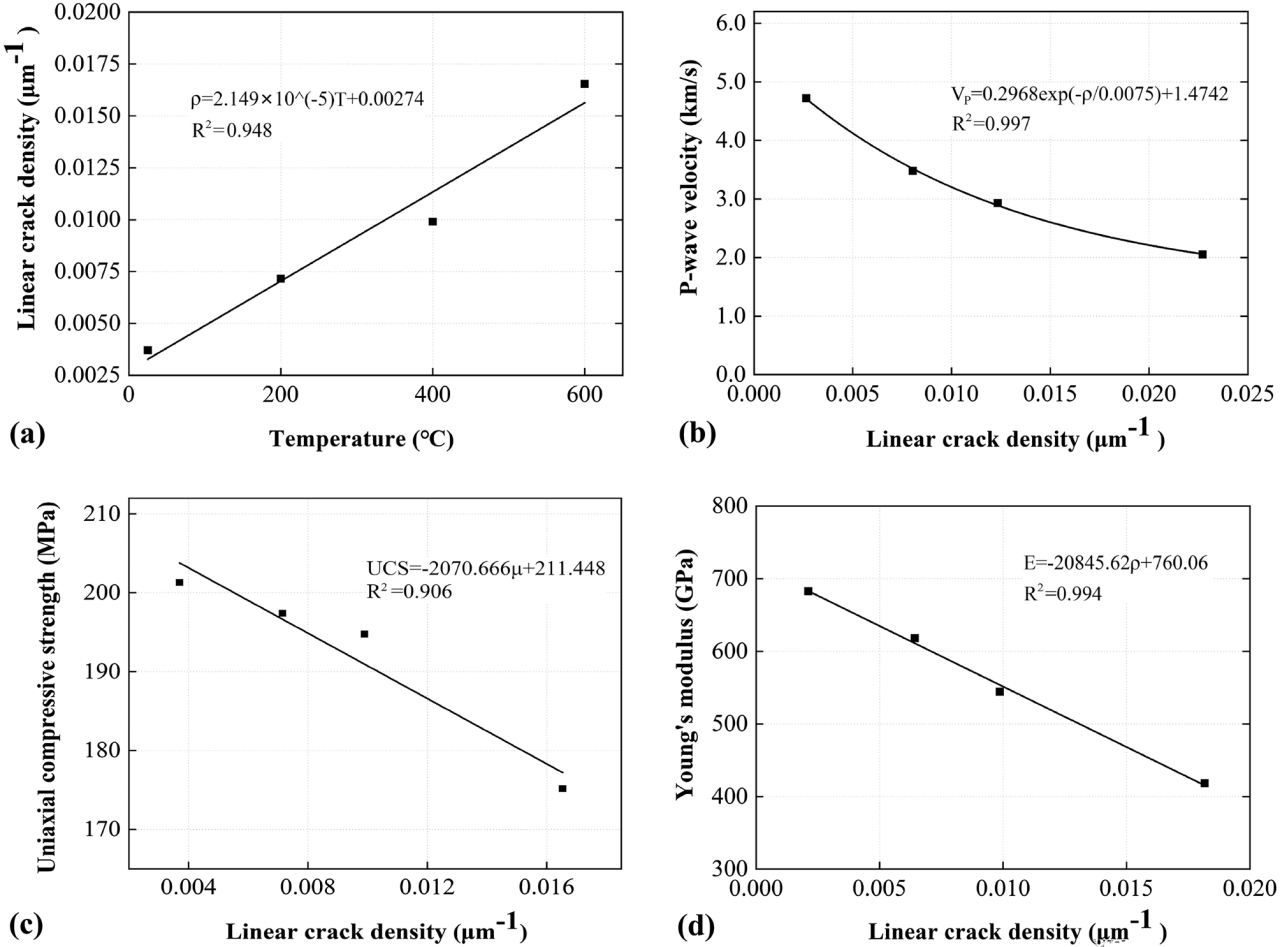
Correlations between (**a**) ρ and treating temperature; (**b**) V_p_ and ρ, (**c**) UCS and ρ; and (**d**) E and ρ.

The ρ of samples treated at 25 °C, 200 °C, 400 °C, and 600 °C are calculated, and the results are expressed in Fig. 9. As shown in Fig. 9a, there exists a linear relationship between ρ with treating temperature (R^2^ of 0.93), increasing from 0.0037 μm^−1^ for the undamaged sample to 0.017 μm^−1^ for sample exposed to 600 °C by 359%. Moreover, the V_p_, UCS and E are all negatively correlated with ρ with high R^2^ (Fig. 9b–d). The results suggest that micro-cracks are a major factor (and probably an intrinsic factor) that governs the macroscopic physical and mechanical behaviour, and ρ may be used as a quantifying index of thermal damage. The physical process behind this is that the intra-granular micro-cracks (thus grain size changes) are generated by thermal loading, these cracks weaken the load-bearing capacity of samples and influence the axial strain during compression. Increases in μ and ρ indicate the formation of more micro-cracks and local crush zones, which compromise the marble integrity and reduce the UCS, E, as well as V_p_. Note that the negative power law was used to correlate ρ and V_p_ in Fig. 9b, since the V_p_ and temperature can be related by a negative power law (as shown in Sect. 3.5), and the ρ and temperature are linearly related. Similar to the previous section, the linear correlation was used as the first step of estimation.

### A novel scheme for thermal damage evaluation

Beijing marble used in this study has been used as a major building material since two thousand years ago; plenty of historic buildings in Beijing are constructed by the Beijing dolomitic marble including four UNESCO cultural heritage sites^3^. However, two of the cultural heritage sites, the Forbidden City (e.g., fires in 1421,1557, 1797, etc.) and the Old Summer Palace (i.e., a fire in 1860), were broke out of fire. For such cases, post-hazard damage evaluation and a satisfactory restoration plan are crucially important but very challenging. The temperatures experienced by different parts of the building are unknown. The amounts of materials allowed for analyses are limited. Engineers also need to replace as few original materials as possible in the restoration process to preserve their historical value and make sure the structures are safe at the same time.

The quantitative correlations sought between the mesoscopic and macroscopic properties of rock (e.g., Figs. 8 and 9) enable one to back-estimate the temperature experienced and to evaluate the levels of thermal damage at different parts of a masonry building caused by, for example, fire hazard with a scientific basis. Based on the results obtained in the present study, a novel scheme of post-fire-hazard damage evaluation of rock is proposed, comprising the following steps:

(I) Classify the types and places of origin of the rock materials used in the given cultural heritage site of interest.
(II) Sample the same type of material from the site and subject them to different high temperatures in the laboratory. Parameters including mineral compositions, PSD, grain size, ρ, UCS, E of each rock type treated under each temperature level are examined.
(III) Analyze the mesoscopic-macroscopic relations and develop correlations between (a) temperature and the scale parameters that define Rayleigh distributions; (b) temperature and ρ; (c) the scale parameters and UCS, axial strain corresponding to the UCS, as well as E; (d) ρ and UCS, strain, as well as E, like those shown in Figs. 8 and 9. For each type of rock, the exact relations may vary due to their own unique mineralogy and bonding conditions.
(IV) (a) Drill small amounts of samples from different areas of the same fire hazard site but with unknown temperatures; (b) conduct XRD, PSD, and photomicrograph analyses to obtain the mesoscopic parameters such as ρ; and (c) map the mesoscopic parameters with the correlations developed in step (III) and then to estimate the temperature experienced by the samples and the macroscopic mechanical properties after thermal damage.
(V) Use the estimated data from step (IV) to calculate the structure stability of the rock samples and devise an appropriate restoration plan accordingly.

Beijing dolomitic marble is taken as an example for the application of the proposed thermal damage evaluation scheme. First of all, the XRD and photomicrograph data need to be determined so as to check if there is any phase change in minerals. If phase change has not been taken place (e.g., treating temperature lower than 600 °C in this example; refer to Table 1 and Fig. 2), then the measured PSD, grain size and ρ are used to evaluate the macroscopic mechanical properties. On the contrary, if phase change has already taken place (e.g., treating temperature higher than 800 °C; refer to Table 1 and Fig. 2), the damage level can be classified according to the mineral types and the percentage of each type of mineral. After obtaining the mineral composition and the percentages via polarized microscope and XRD analyses, the maximum temperature experienced by each sample is back-calculated by comparing the mineral composition of heated samples with that of unheated samples and also conducting chemical equation calculation based on the decomposition temperatures of each rock-forming mineral.

The proposed scheme may also be used for the quality evaluation of rocks used for thermal energy storage. It should be pointed out that the proposed scheme is based on the results obtained from the classifications and trans-scale correlations of one type of monomineralic rock. In principle, however, this scheme should also be applicable to polymineralic rocks, but certainly, more parameters and correlations need to be determined to modify steps (II) and (III).

## Conclusions

In this study, effects of different high temperatures on a monomineralic marble, which consists of over 98% dolomite and is also a major construction material of UNESCO cultural heritage sites in Beijing, were studied. Thermally-induced changes in both the mesoscopic and macroscopic properties of the rock were examined. Based on the test results, the following conclusions were drawn:

1. High temperature treatments led to micro-crack initiation and propagation in the marble samples. The evolution of micro-crack development can be classified into three stages: initial stage (25 °C–400 °C treatment) with limited to moderate micro-crack development, transitional stage (600 °C treatment) with significant micro-crack development, and saltational stage (800 °C–1000 °C treatment) with drastic micro-crack development. Intra-granular micro-cracks and grain crushes were mainly observed after 600 °C treatments and the above. Mineral decomposition is observed for samples treated after 800 °C and 1000 °C. The micro-crack development and mineral decomposition led to a deterioration of the physico-mechanical properties, namely, an increase in open porosity, weight loss and strain corresponding to the peak stress but a decrease in P-wave velocity, uniaxial compressive strength, Young’s modulus and brittleness.
2. Thermally-induced changes in the macroscopic properties of the marble were closely related to those in the mesoscopic parameters. Higher temperature led to more severe grain crushes and more inter-granular and intra-granular micro-cracks, thus causing higher linear crack density and a higher percentage of small size particles. Hence several trans-scale relationships for the marble were derived. The thermally-induced evolution of particle size can be characterized by modified Rayleigh distributions, in which the scale parameter is proportional to the treating temperatures. Linear crack density and scale parameter can be used as effective mesoscopic indices to estimate thermally-induced changes in the macroscopic parameters including compressive strength and Young’s modulus. The XRD and chemical-equation-based relative atomic mass calculation are useful measurement tools to estimate the degrees of mineral phase change when treated above the critical temperature.
3. Based on the XRD based mineral decomposition criteria and trans-scale relationships, a novel thermal damage evaluation scheme for monomineralic rocks such as the marble was proposed. This scheme is useful in cases that only limited amounts of samples are available or the sample sizes are normally small, such as post-fire-hazard damage evaluation of masonry buildings and quality evaluation of materials used for thermal energy storage.

## Materials and methods

### Materials

In this study, a fine-grained Beijing dolomitic marble was selected for investigation due to its purity (i.e., composed of almost only dolomite) and engineering significance (i.e., used as a material for historic buildings). As micro-crack development characteristics (e.g., length and orientation of micro-cracks) in thermal loading can be influenced by the type of rock-forming minerals^7^, it is preferred to select a material with the simplest mineral constituent for pioneered quantitative studies. The Beijing marble is a single-phase dolomitic rock with no obvious lattice preferred orientation (LPO) and shape preferred orientation (SPO)^3^, which has an advantage for variable control and analysis simplification. Besides, considering from an engineering perspective, there are plenty of historic buildings constructed by the Beijing dolomitic marble including four UNESCO cultural heritage sites: the Forbidden City, the Temple of Heaven, the Summer Palace, and the Thirteen Ming Tombs^3^. Thus, the material is worth careful investigation and the results obtained would make direct contributions to engineering practice.

The examined marble was quarried from one of the historical quarries in Dashiwo county, Beijing’s Fangshan District, China. Limited to 1.6–1.4 Ga, the strata producing Beijing dolomitic marble belong to the Wumishan carbonate formation of the Jixianian system (Jx-Wumishan Fm) in litho-choronostratigraphic units^42^. The marble, which is composed of mostly dolomite with minor portions of quartz and clay minerals, is white in color and semi-transparent under flashlight. No obvious crystal orientation was observable from thin sections of this rock. The grain size ranged from 5 to 451 μm with an average of 85.67 μm (based on an analysis of 1476 particles). To minimize sample-to-sample variability, a marble block with a dimension of approximately 600 × 400 × 150 mm^3^ was carefully selected and transported to the laboratory. The selected block has no impurity and defects visible to naked eyes. All samples used for examination were cored from this block to ensure that they experienced the same geological process, thus their initial properties are the same in both mesoscopic and macroscopic scales. The samples were prepared according to different experimental purposes (Table 3), placed in an air-conditioned room (around 25 °C, 35% relative humidity) for a week, and eventually dried in an electronic oven at 65 °C for 48 h. After the drying process, the samples were stored in plastic bags and sealed prior to testing.

**Table 3:** Shape and dimension of samples used for different experimental purposes.

Shape	Dimension (mm)	No. of samples	Parameters
Cylinder	D × H, 50 × 100	6	P-wave velocity, uniaxial compression
Cylinder	D × H, 10 × 15	6	Mercury intrusion porosimetry
Cube	50 × 50 × 50	6	Weight loss, X-ray diffraction
Pie	D × H, 50 × 30	6	Used to prepare thin sections
Thin section	20 × 50 × 1	6	Optical microscope observation

Note that all samples were prepared from the same marble block. The symbol D and H denotes diameter and height, respectively.

### Thermal treatments

A programmable heating device (model: SG-XL1200, SIOM (CAS), China) was used to apply controlled thermal treatments to the rock samples. The heating device has a maximum operating temperature of 1200 °C. In the experiments, different rock samples were heated to 25 °C, 200 °C, 400 °C, 600 °C, 800 °C, and 1000 °C (denoted as samples M25, M200, M400, M600, M800, and M1000, respectively). Each sample was heated to the target temperature at a rate of 1 °C/min and the temperature was maintained for five hours to let the sample fully bake. The samples were then cooled down to approximately 200 °C, also at a rate of 1 °C/min. Since the cooling rate became less than 1 °C/min when the temperature dropped below 200 °C, the device was switched off and the samples were left in the device for 12 h to reach the room temperature of 25 °C. The rates were chosen to be equal to or lower than 1 °C/min to prevent any “thermal shock” effect (i.e., a damaging effect related to the rate of change of temperature)^16,17,43^. After subjecting to a cycle of heating and cooling, the samples were sealed in plastic bags again to prevent humidity intrusion.

### Testing equipment and procedures

After the thermal treatments, the samples of different shapes and dimensions (Table 3) were subjected to tests in both mesoscopic scale (i.e., X-ray diffraction tests, Mercury intrusion porosimetry tests, Optical microscope observation) and macroscopic scale (Weight loss tests, P-wave velocity measurement, Uniaxial compressive tests). As mentioned in the previous section, the objectives of the present study are to characterize the effect of thermal loading on physico-mechanical properties of tested material from different scales and seek intrinsic mesoscopic-macroscopic relationships quantitatively. Therefore, all of the tests were carefully performed using top-tier experiment instruments to minimize instrument-induced error and ensure data reliability. The detailed procedures of conducted tests are provided as follows:

#### X-ray diffraction tests (XRD)

The mineral compositions of each cubic sample were determined using the powder X-ray diffraction method. A diffractometer (model: D/max 2400, Rigaku Corporation, Japan) with Cu radiation was used. Before analysis, each cubic sample was grounded into powders and sieved through a size of approximately 70 microns. The testing condition was set as: 1° divergence slit, 1° anti-scatter slit, 0.3 mm receiving slit, 0.02°/step length, and 8°/min.

#### Mercury intrusion porosimetry (MIP) tests

**Figure 10:**
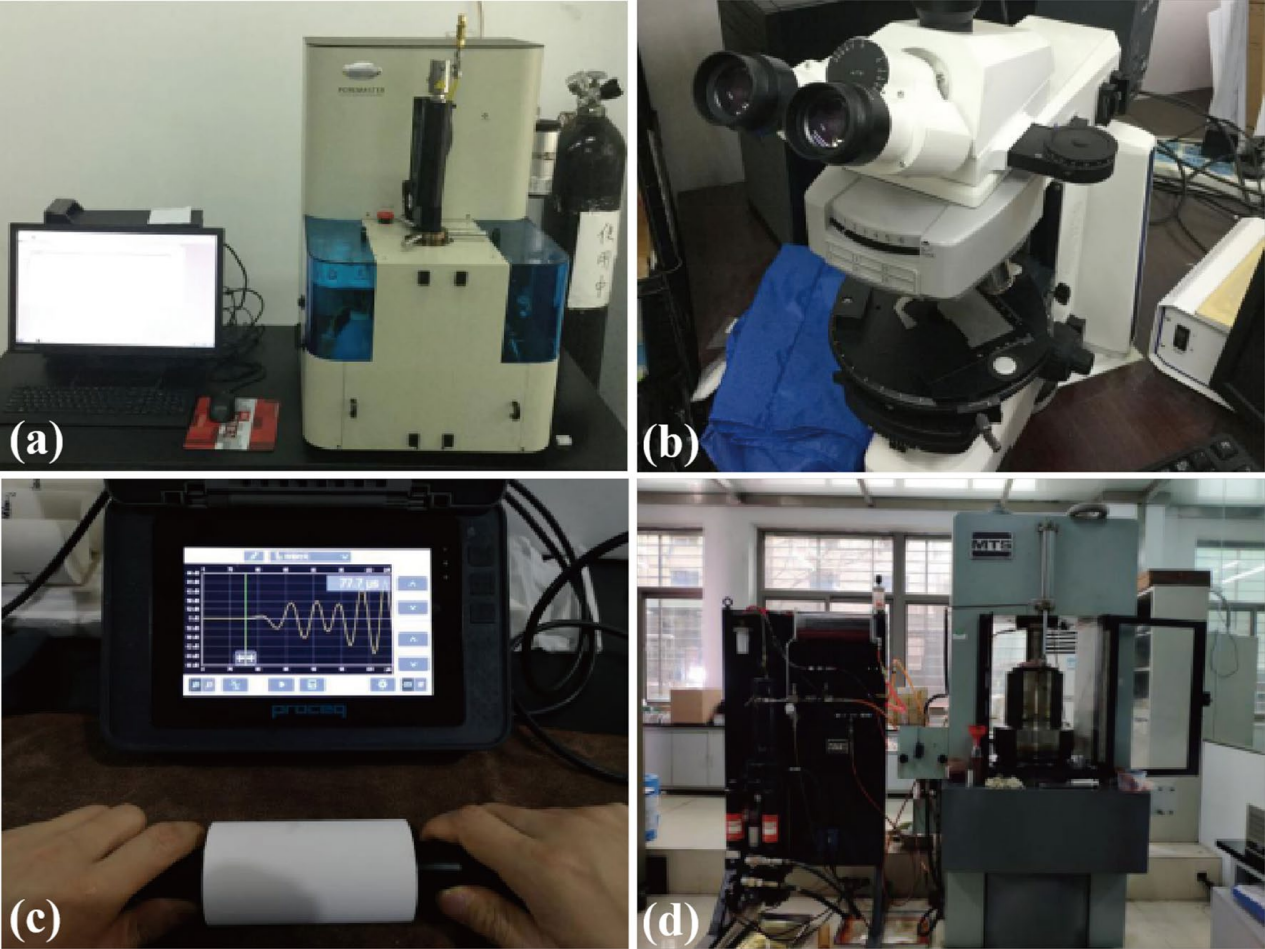
Testing equipment used in the study: (**a**) automatic mercury porosimeter (PoreMaster 60, Quantachrome Corporation, USA), (**b**) polarizing microscope (Axio Scope A1, Carl Zeiss Corporation, Germany), (**c**) ultrasonic pulse velocity testing equipment (Pundit PL-200, Proceq Group, Switzerland), and (**d**) MTS-815.03 rock testing system (MTS Systems Corporation, USA).

A mercury porosimeter (model: PoreMaster 60, Quantachrome Corporation, USA) was used to measure the pore size distribution and open porosity of each cylindrical sample (Fig. 10a). The maximum pressure applied was 4000 PSI (approximately 27.6 MPa).

#### Optical microscope observation

After the thermal treatments, the pie-shaped samples were immersed in resin adhesive and cut into thin sections. The thickness of thin section was chosen to be 1 mm to minimize any damage induced during sample preparation. The observation was performed by using a microscope (model: Axio Scope A1, Carl Zeiss Corporation, Germany) set in the reflection mode and plane-polarized light (Fig. 10b). Photomicrographs of each of the thin sections were captured by the built-in camera.

#### Weight loss tests

Each cubic sample was weighed before and after the applied heating–cooling cycle. Any weight loss was expressed by the weight difference before and after heat treatment over the weight before heat treatment.

#### P-wave velocity measurement

P-wave velocities (V_p_) of cylindrical rock samples were measured by using an ultrasonic pulse velocity testing equipment (model: Pundit PL-200, Proceq Group, Switzerland) before and after the thermal treatments (Fig. 10c). White vaseline was used as a coupling agent. The V_p_ was measured by ultrasonic transducers at a frequency of 250 kHz. In each test, five measurements were made, and three data with the least discrepancies were chosen for subsequent analysis.

#### Uniaxial compressive tests

The tests were conducted by a rock testing system (model: MTS-815.03, MTS Systems Corporation, USA) (Fig. 10d) after the thermal treatments. To study the post-peak response of samples treated at different temperatures, axial displacement-controlled loading at a rate of 0.05 mm/min was applied until the stress–strain curve reached a stable post-peak stage. The axial stress and strain were recorded during the entire loading process. The uniaxial compressive strength (UCS) and Young’s modulus (E) were determined from each measured stress–strain curve.

### Improved image processing techniques

**Figure 11:**
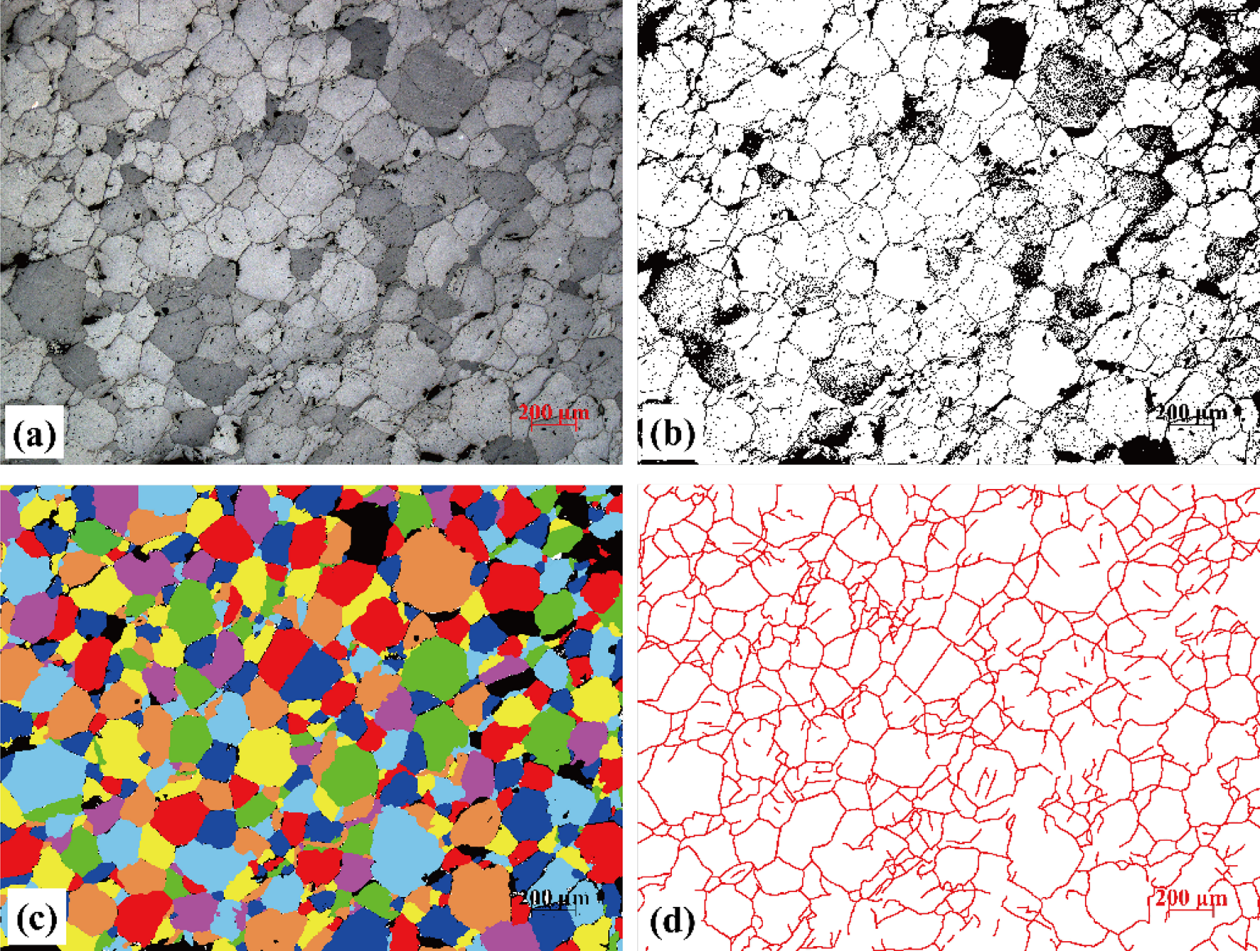
Improved image processing procedures for mesoscopic analysis of images taken from the optical microscope observation tests of a rock sample after 600 °C thermal treatment. The original image (**a**) was reverse-segmented and converted to grey image (**b**), in which the grains were filled with different colors (**c**). Image (**d**) shows the curves that represent the micro-cracks in image (**a**).

Analyzing mesoscopic images obtained from the optical microscope observation tests could be challenging. As shown in Fig. 11a, the contrast between the boundaries and grain minerals captured in the reflection mode was weak (i.e., the color of cracks is only slightly darker than that of grains). Traditional image processing techniques, such as adjusting the contrast and brightness, transforming into gray level image, then segmenting the grains by grade level or by RGB, were not effective in separating the grains accurately. To improve the accuracy of the image analysis, a multi-step approach was adopted to segment grain minerals and to extract micro-crack network information. Figure 11 shows the proposed image processing procedures. To ensure that the results are comparable, the twelve photos taken from M25, M200, M400, and M600 (three images each) used for analysis were set to have the same size and amplification factors in the optical microscope observation.

After adjusting the brightness and contrast, the boundaries of minerals in the original image were sharpened. The image was imported into the PCAS software^44^. The boundary network was extracted using the Reverse Segmentation module and then converted into a gray image (Fig. 11b). By using Photoshop software, grains in each binary image were filled with different colors. After extracting and enhancing the mineral boundaries, the Quick Selection Tool in the software was used to accurately and effectively select grains from thousands of them in a sample. In cases where grains (typically those with gradations of grey and blurry boundaries) cannot be automatically identified, the original image was checked (Fig. 11a) and the grains were filled manually. Finally, the equivalent diameter of each grain (i.e., an equivalent diameter corresponding to the area of a grain) in the color-filled image (Fig. 11c) were calculated using the PCAS software.

Linear crack density is an important parameter to evaluate thermal damage of rocks^40^. The automatic identification of micro-cracks by a software often has a drawback of mis-identification^20^. In this study, a novel tracing and calculation method was developed. First, a new image layer on top of the photomicrograph was created in the Photoshop. The cracks were then traced manually and exported as a crack figure (Fig. 11d). The crack figure was used to calculate the total area of the curves that represent the cracks in that image. The total crack length is represented by the total area of the cracks divided by the crack width. It should be noted that in this method, the width of curves that represent cracks in Fig. 11d should be properly defined according to the proportion of the size of photomicrograph and the width of tracing curves to ensure the calculation accuracy. The scale of photomicrograph was in microns, the width of the curves was set to be 6.7 μm.

## Supplementary Information

Supplementary Figures.

## Data Availability

Some or all data, models, or code that support the findings of this study are available from the corresponding authors upon reasonable request.

## References

[1] Koca MY (2006). Changes in the engineering properties of marble in fire-exposed columns. Int. J. Rock Mech. Min. Sci..

[2] Martinho E, Mendes M, Dionísio A (2017). 3D imaging of P-waves velocity as a tool for evaluation of heat induced limestone decay. Constr. Build. Mater..

[3] Liu J, Zhang Z, Li B (2019). Microscopic & macroscopic characterizations of Beijing marble as a building material for UNESCO heritage sites: New insights into physico-mechanical property estimation and weathering resistance. Constr. Build. Mater..

[4] Murru A, Freire-lista DM, Fort R, Varas-muriel MJ, Meloni P (2018). Evaluation of post-thermal shock effects in Carrara marble and Santa Caterina di Pittinuri limestone. Constr. Build. Mater..

[5] Cheng Y, Wong LNY (2018). Microscopic characterization of tensile and shear fracturing in progressive failure in marble. J. Geophys. Res. Solid Earth.

[6] Liu J, Li B, Tian W, Wu X (2018). Investigating and predicting permeability variation in thermally cracked dry rocks. Int. J. Rock Mech. Min. Sci..

[7] Li Z, Wong LNY, Teh CI (2020). Influence of thermal and mechanical loading on development of microcracks in granite. Rock Mech. Rock Eng..

[8] Zhang S, Wen Z, Wang G, Lou G, Liu X (2021). Kinetic analyses of coke combustion and thermal decompositions of limestone and dolomite based on the sintering atmosphere. Fuel.

[9] Zhang W, Sun Q, Hao S, Geng J, Lv C (2016). Experimental study on the variation of physical and mechanical properties of rock after high temperature treatment. Appl. Therm. Eng..

[10] Yao M, Rong G, Zhou C, Peng J (2016). Effects of thermal damage and confining pressure on the mechanical properties of coarse marble. Rock Mech. Rock Eng..

[11] Wong LNY, Li Z, Kang HM, Teh CI (2017). Dynamic loading of carrara marble in a heated state. Rock Mech. Rock Eng..

[12] Yin W, Feng Z, Zhao Y (2021). Effect of grain size on the mechanical behaviour of granite under high temperature and triaxial stresses. Rock Mech. Rock Eng..

[13] Zhang Z, Liu J, Li B, Yang X (2018). Thermally induced deterioration behaviour of two dolomitic marbles under heating-cooling cycles. R. Soc. Open Sci..

[14] Cantisani E (2009). Thermal stress in the Apuan marbles: Relationship between microstructure and petrophysical characteristics. Int. J. Rock Mech. Min. Sci..

[15] Meng T, Liu R, Meng X, Zhang D, Hu Y (2019). Evolution of the permeability and pore structure of transversely isotropic calcareous sediments subjected to triaxial pressure and high temperature. Eng. Geol..

[16] Zhou C, Wan Z, Zhang Y, Gu B (2018). Experimental study on hydraulic fracturing of granite under thermal shock. Geothermics.

[17] Kumari WGP, Ranjith PG, Perera MSA, Chen BK, Abdulagatov IM (2017). Temperature-dependent mechanical behaviour of Australian Strathbogie granite with different cooling treatments. Eng. Geol..

[18] Freire-lista DM, Fort R, Varas-muriel MJ (2016). Thermal stress-induced microcracking in building granite. Eng. Geol..

[19] Sun Q (2016). Thermal properties of sandstone after treatment at high temperature. Int. J. Rock Mech. Min. Sci..

[20] Griffiths L, Heap MJ, Baud P, Schmittbuhl J (2017). Quantification of microcrack characteristics and implications for stiffness and strength of granite. Int. J. Rock Mech. Min. Sci..

[21] Zhu Z, Tian H, Jiang G, Cheng W (2018). Effects of high temperature on the mechanical properties of Chinese marble. Rock Mech. Rock Eng..

[22] Biró A, Hlavička V, Lublóy É (2019). Effect of fire-related temperatures on natural stones. Constr. Build. Mater..

[23] Malaga-Starzec K, Åkesson U, Lindqvist JE, Schouenborg B (2006). Microscopic and macroscopic characterization of the porosity of marble as a function of temperature and impregnation. Constr. Build. Mater..

[24] Karaca Z, Hacimustafaoʇlu R, Gökçe MV (2015). Grain properties, grain-boundary interactions and their effects on the characteristics of marbles used as building stones. Constr. Build. Mater..

[25] Peng J, Rong G, Cai M, Zhou CB (2015). A model for characterizing crack closure effect of rocks. Eng. Geol..

[26] Cai M (2004). Generalized crack initiation and crack damage stress thresholds of brittle rock masses near underground excavations. Int. J. Rock Mech. Min. Sci..

[27] Peng J, Rong G, Cai M, Yao M, Zhou C (2016). Comparison of mechanical properties of undamaged and thermal-damaged coarse marbles under triaxial compression. Int. J. Rock Mech. Min. Sci..

[28] Wen T, Tang H, Ma J, Wang Y (2018). Evaluation of methods for determining crack initiation stress under compression. Eng. Geol..

[29] Su H (2017). Strength and deformation behaviors of veined marble specimens after vacuum heat treatment under conventional triaxial compression. Acta Mech. Sin. Xuebao.

[30] Liu S, Xu J (2015). Analysis on damage mechanical characteristics of marble exposed to high temperature. Int. J. Damage Mech..

[31] Caceres PG, Attiogbe EK (1997). Thermal decomposition of dolomite and the extraction of its constituents. Miner. Eng..

[32] Escardino A, Garcia-Ten J, Feliu C (2008). Kinetic study of calcite particle (powder) thermal decomposition: Part I. J. Eur. Ceram. Soc..

[33] Chen YL, Wang SR, Ni J, Azzam R, Fernández-steeger TM (2017). An experimental study of the mechanical properties of granite after high temperature exposure based on mineral characteristics. Eng. Geol..

[34] Sun H, Sun Q, Deng W, Zhang W, Lü C (2017). Temperature effect on microstructure and P-wave propagation in Linyi sandstone. Appl. Therm. Eng..

[35] Peng R (2014). Energy dissipation and release during coal failure under conventional triaxial compression. Rock Mech. Rock Eng..

[36] Gautam PK (2021). Damage characteristics of jalore granitic rocks after thermal cycling effect for nuclear waste repository. Rock Mech. Rock Eng..

[37] Miao S, Pan PZ, Zhao X, Shao C, Yu P (2021). Experimental study on damage and fracture characteristics of Beishan granite subjected to high-temperature treatment with DIC and AE techniques. Rock Mech. Rock Eng..

[38] Kurth RE, Cox DC (1985). Discrete probability distributions for probabilistic fracture mechanics. Risk Anal..

[39] Kumari WGP, Ranjith PG, Perera MSA, Chen BK (2018). Experimental investigation of quenching effect on mechanical, microstructural and flow characteristics of reservoir rocks: Thermal stimulation method for geothermal energy extraction. J. Pet. Sci. Eng..

[40] Rong G, Peng J, Yao M, Jiang Q, Wong LNY (2018). Effects of specimen size and thermal-damage on physical and mechanical behavior of a fine-grained marble. Eng. Geol..

[41] Pappalardo G, Mineo S, Monaco C (2016). Geotechnical characterization of limestones employed for the reconstruction of a UNESCO world heritage Baroque monument in southeastern Sicily (Italy). Eng. Geol..

[42] Gao LZ, Ding XZ, Gao Q, Zhang CH (2010). New geological time scale of Late Precambrian in China and geochronológy. Geol. China.

[43] Hall K, André MF (2001). New insights into rock weathering from high-frequency rock temperature data: An Antarctic study of weathering by thermal stress. Geomorphology.

[44] Liu C, Tang CS, Shi B, Suo W (2013). Automatic quantification of crack patterns by image processing. Comput. Geosci..

