# Supplementary material for: Mesoscopic and macroscopic investigation of a dolomitic marble subjected to thermal damage
Source: Sci Rep. 2022 Sep 12;12:15308. doi: 10.1038/s41598-022-19655-x (PMC9468164; doi:10.1038/s41598-022-19655-x)
Supplement: Supplementary file 1 — Supplementary Figures. [file 41598_2022_19655_MOESM1_ESM.pdf]

Supplementary materials

The followings are fitted plots with modified Rayleigh distribution calculated by SPSS:

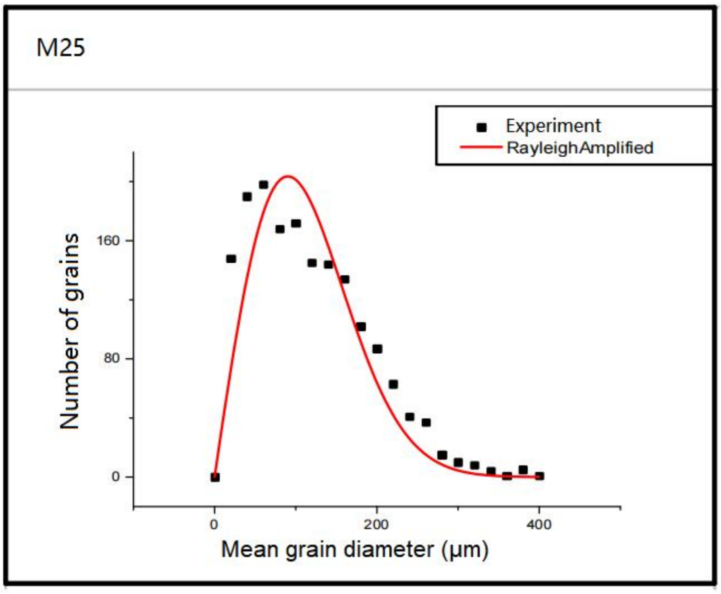

Fig. S1 Fitted plot of M25 data.

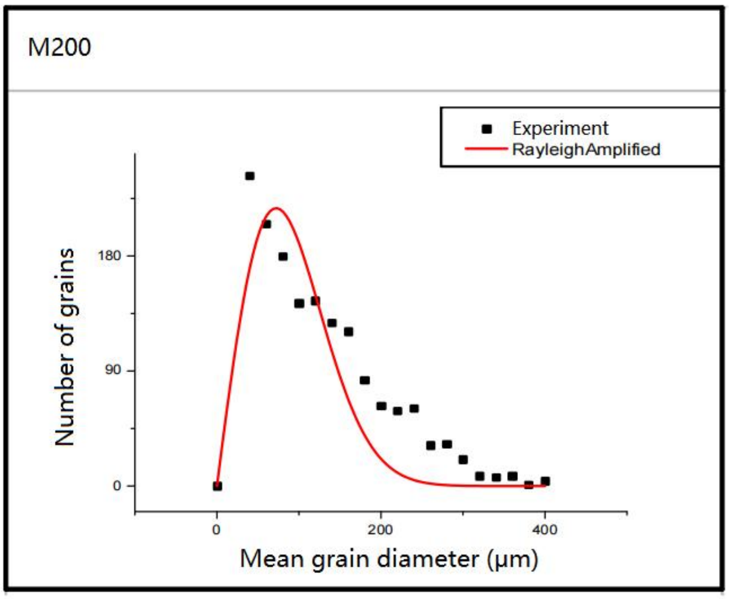

Fig. S2 Fitted plot of M200 data.

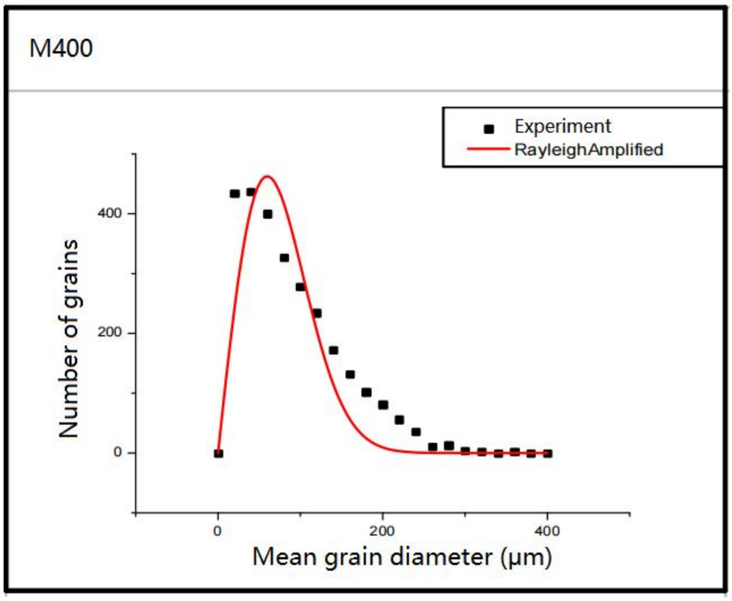

Fig. S3 Fitted plot of M400 data.

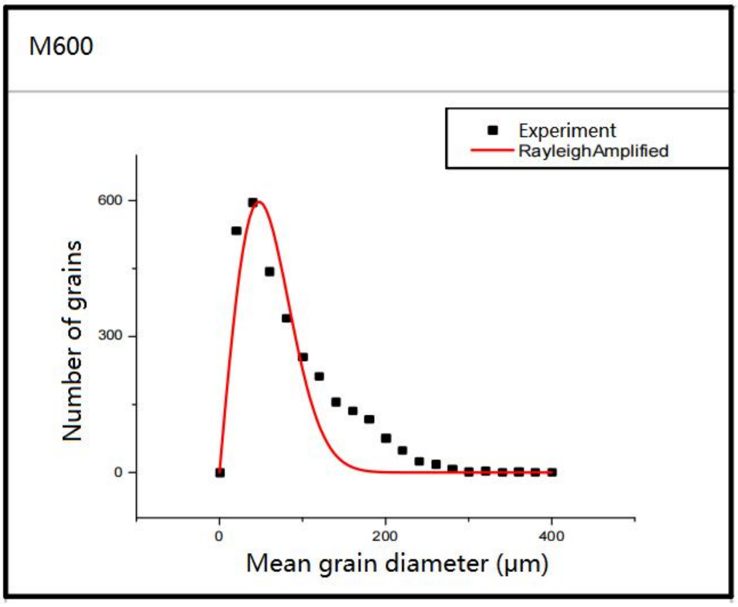

Fig. S4 Fitted plot of M600 data.
